# Unveiling the genome of a high-risk pandrug-resistant *Klebsiella pneumoniae* emerging in the Brazilian Amazon Region, 2022

**DOI:** 10.1590/0074-02760230081

**Published:** 2023-10-27

**Authors:** Érica Lourenço Fonseca, Sérgio M Morgado, Fernanda S Freitas, Nathalia S Bighi, Rosângela Cipriano, Ana Carolina Paulo Vicente

**Affiliations:** 1 Fundação Oswaldo Cruz-Fiocruz, Instituto Oswaldo Cruz, Laboratório de Genética Molecular de Microrganismos, Rio de Janeiro, RJ, Brasil; 2 São Domingos Hospital, São Luís do Maranhão, MA, Brasil

**Keywords:** PDR, tigecycline resistance, colistin resistance, ompK, acrAB, untreatable bacteria

## Abstract

**BACKGROUND:**

Pandrug-resistant (PDR) *Klebsiella pneumoniae* has been reported sporadically in many countries and remains rare in Brazil.

**OBJECTIVES:**

This study unravelled the genetic determinants involved with the PDR background of a clinical ST11 *K. pneumoniae* recovered in the Brazilian Amazon Region, where *K. pneumoniae* genomic and epidemiological information is scarce.

**METHODS:**

Kp196 was submitted to the antimicrobial susceptibility test by the disk-diffusion method and minimum inhibitory concentration (MIC) determination. The whole genome sequencing was obtained and the sequence type was determined by core genome multilocus sequence typing (cgMLST). Its intrinsic and acquired resistome was assessed by Comprehensive Antibiotic Resistance Database (CARD) and comparison with wild-type genes.

**FINDINGS:**

The analyses revealed that Kp196 belonged to the pandemic ST11 and presented the PDR phenotype. Its acquired resistome was composed of a huge set of clinically relevant resistance determinants, including *bla*_CTX-M-15_ and *bla*_NDM-1_, all found in the vicinity of mobile platforms. Considering its intrinsic resistome, the multidrug resistance, especially to colistin, tigecycline and fluoroquinolones, was multifactorial and attributed to modifications (indels, missense mutations, and gene disruption) in several housekeeping genes (*arnT*/*phoQ/mgrB/ramR/acrB/gyrA/parC/ompK35-36-37*). The Kp196 intrinsic resistome was also observed in a ST11 environmental strain, although harbouring distinct acquired resistomes.

**CONCLUSIONS:**

An accumulation of different resistance mechanisms regarding the intrinsic resistome accounts for a more stable resistome, strongly contributing to the Kp196 PDR phenotype.

Pandrug resistance (PDR) is related to the non-susceptibility to all agents in all antimicrobial categories considered approved and useful for treating an infection caused by a specific organism.^1^ *Klebsiella pneumoniae* is featured by a remarkable propension for multidrug resistance acquisition, and infections caused by multidrug- (MDR) and extensively drug-resistant (XDR) strains are highly prevalent worldwide, while PDR remains rare.^2^ These MDR/XDR lineages are frequently carbapenem-resistant, and in this case, tigecycline and colistin remain the unique effective therapeutic choices.^3^ Therefore, tigecycline and colistin co-resistance in carbapenem-resistant *K. pneumoniae* may result in apparently untreatable organisms, leading to a worrisome impact on clinical outcomes. Eventually, strains of the international high-risk clonal complex CC258 (ST11, ST437, and ST258) have presented the PDR profile. In Brazil, so far, PDR *K. pneumoniae* has only been reported in a few CC258 strains in the Southeast Brazilian Region,^4^ ^,^ ^5^ and the genomic features involved with the PDR manifestation were rarely assessed. In fact, a recent study from our group demonstrated that most of the publicly available *K. pneumoniae* genomes in Brazil were obtained from the South/Southeast regions (n = 310), while only 62 genomes from the North/Northeast regions were available to date.^6^

The present study unravelled the genome of a clinical PDR *K. pneumoniae* strain, Kp196, belonging to the high-risk pandemic ST11. This strain was recovered in Maranhão, a Northeast Brazilian State of the eastern Amazon Region, where *K. pneumoniae* genomic information is scarce and the epidemiological scenario is poorly understood. The main genetic determinants involved with Kp196 PDR background were revealed, and this genome was compared to the unique available genome from Maranhão, KPCEU1 (**GCA_018335415.1**), which also belonged to the ST11 but was recovered from the environment (mangrove). In this way, this study not only contributed to the increment of the genomic information concerning the mechanisms involved with PDR emergence but also to the understanding of the epidemiological scenario of high-risk *K. pneumoniae* lineages in an underrepresented Brazilian region (Amazon Region).

## MATERIALS AND METHODS

In 2022, the Kp196 was recovered from the tracheal secretion of an inpatient of the Djalma Marques Hospital, the largest public hospital in São Luís city (Maranhão) providing urgent and emergency care, which is located in the eastern Amazon Region.

The antimicrobial susceptibility test (AST) was determined for all antibiotics considered for *Enterobacteriaceae* resistance classification,^1^ and interpreted according to the Clinical and Laboratory Standards Institute (CLSI),^7^ and European Committee on Antimicrobial Susceptibility Testing (EUCAST) (for tigecycline and polymyxins) guidelines.^8^

The Kp196 genome was obtained on the Illumina Hiseq 2500 using Nextera paired-end kit for library construction. SPAdes assembler v3.15.2 was used for genome assembling,^9^ and gene prediction/annotation was conducted with Prokka v1.14.6.^10^ Core genome multilocus sequence typing (cgMLST) was determined in the Bacterial Isolate Genome Sequence Database (BIGSdb; http://bigsdb.pasteur.fr/klebsiella/). The Comprehensive Antibiotic Resistance Database (CARD) was used for antimicrobial resistance gene (ARG) prediction.^11^ Plasmid replicon identification was conducted with the PlasmidFinder.^12^ The deduced protein of each Kp196 chromosomal gene involved with resistance was compared with that of the wild-type reference strains *K. pneumoniae* NTUH-K2044 (**NC_012731.1**) and MGH 78578 (**CP000647**). The Kp196 genome sequence was deposited in the GenBank under accession no. **JAQOSS000000000** and with BioProject no. **PRJNA926954**.

The *K. pneumoniae* KPCEU1 genome (**GCA_01833-5415.1**) was recovered in 2020 from the sediment of the Anil River, localised in São Luís, Maranhão. This was the unique other *K. pneumoniae* genome reported to date in Maranhão. Since KPCEU1 also belonged to ST11 and given its environmental nature, Kp196 was directly compared to KPCEU1 genome to assess the clonal relationship and the particularities of the resistomes of two contemporary ST11 genomes, recovered from the same city but from distinct sources (clinical and environmental). For this analysis, a single nucleotide polymorphisms (SNPs)-based genetic reconstruction was performed including 12 publicly available ST11 *K. pneumoniae* genomes previously recovered in Brazil, and an ST340 genome as an outgroup. The core genome was determined by Roary v3.13.0^13^ and sites of the core genes with SNPs were extracted using snp-sites v2.5.1.^14^ The phylogeny was performed with IQTree v1.6.12.^15^ The KPCEU1 genome was also submitted to resistome mining by using the CARD tool.

## RESULTS AND DISCUSSION

The *in vitro* analyses revealed that Kp196 corresponded to a PDR strain (Table I), and the cgMLST assigned it to the ST11 pandemic lineage. In spite of the high prevalence of this lineage in Brazil,^6^ this is the first report of a PDR ST11 in the country. In fact, PDR *K. pneumoniae* remains rare in Brazil, having only been reported in ST437 and ST258 restricted to the South/Southeast regions.^4^ ^,^ ^5^ ^,^ ^16^

**TABLE I:** Kp126 pandrug-resistant (PDR) phenotype

	MIC (mg/L)									Resistance profile determined by disc-diffusion^c^
	IPM^a^	MEM^a^	ETP^a^	DOR^a^	CZA^a^	C/T^a^	TGC^a^	CST^b^	PMB^b^	
Kp196	> 32	> 32	> 32	> 32	> 256	> 256	0.75	8	4	GEN, TOB, AMK, NET, CPT, TIM, TZP, CFZ, CXM, CTX, CAZ, FEP, FOX, CTT, CIP, SXT, ATM, AMP, AMC, SAM, CHL, FOF, TET, DOX, MIN

*a*: minimum inhibitory concentration (MIC) determined by E-Test method.^7^ The new tigecycline breakpoints for resistance (> 0.5 mg/L) recently revised by EUCAST were applied.^8^ ^)^ *b*: MIC determined by broth microdilution method. Colistin and polymyxin B MIC breakpoints for resistance > 2 mg/L.^8^ ^)^ *c*: antimicrobial susceptibility test (AST) determined by disk-diffusion method.^7^ IPM: imipenem; MEM: meropenem; ETP: ertapenem; DOR: doripenem; CZA: ceftazidime/avibactam; C/T: ceftolozane/tazobactam; TGC: tigecycline; CST: colistin; PMB: polymyxin B; GEN: gentamicin; TOB: tobramycin; AMK: amikacin; NET: netilmicin; CPT: ceftaroline; TIM: ticarcillin/clavulanic acid; TZP: piperacillin/tazobactam; CFZ: cafazolin; CXM: cefuroxime; CTX: cefotaxime; CAZ: ceftazidime; FEP: cefepime; FOX: cefoxitin; CTT: cefotetan; CIP: ciprofloxacin; SXT: trimethoprim/sulfamethoxazole; ATM: aztreonam; AMP: ampicillin; AMC: amoxacillin/clavulanic acid; SAM: ampicillin/sulbactam; CHL: chloramphenicol; FOF: fosfomycin; TET: tetracycline; DOX: Doxycycline; MIN: minocycline.

The PDR phenotype was in accordance with the Kp196 resistome, which was composed of genes associated with resistance to aminoglycosides (*aadA1*, *aacA4*, *strAB*, *aph(3’)-VI*, *aac(3)-IId*), fluoroquinolones (*qnrS1*, *qnrB1*, *oqxAB*), trimethoprim (*dfrA14*), sulfonamides (*sul2*), tetracycline (*tetD*), fosfomycin (*fosA5*), chloramphenicol (*catB3*) and β-lactams including carbapenems (*bla*_SHV-11_, *bla*_CTX-M-15_, *bla*_OXA-9_, *bla*_OXA-1,_ bla_TEM-1,_ *bla*_NDM-1_). Although less prevalent than the carbapenemase-coding *bla*_KPC-2_ gene, several studies demonstrated the occurrence of *bla*_NDM-1_ among clinical *K. pneumoniae* in Brazil,^17^ including the ST11 lineage, where this gene was found in the context of an IncC plasmid.^18^ Interestingly, the *bla*_NDM-1_ occurrence in Brazil was not restricted to clinical settings since it had already been found in environmental *K. pneumoniae* isolates recovered from both surface waters,^19^ and wastewater treatment plants (WWTPs).^20^

Most of the genes composing the resistome were flanked or in the vicinity of insertion sequences and plasmid-related genes, the exception was the *tetD*, *fosA5*, and *bla*_SHV-11_, which were chromosomally encoded. In fact, Kp196 harboured *repA*, *repB* and *repE* genes from IncFIB and IncR plasmids.

**Fig. 1:**
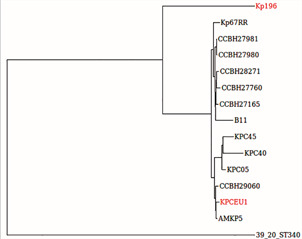
single nucleotide polymorphisms (SNPs)-based genetic tree of ST11 genomes from Brazil. The Kp196 and KPCEU1 genomes are highlighted.

**Fig. 2:**
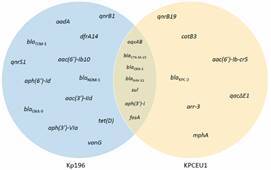
Venn diagram of ST11 Kp196 (blue) and KPCEU1 (yellow) resistomes highlighting the shared and particular antibiotic resistance genes.

The genomic information of *K. pneumoniae* circulating in the Amazon Region is scarce: only 26 genomes in the western part (12 genomes from Amazonas and 14 genomes from Roraima)^6^ and two in the eastern part (one from Pará and one from Maranhão) had already been published. Interestingly, this unique publicly available genome from Maranhão (KPCEU1) corresponded to a strain also belonging to ST11 but recovered from an environmental source. In this way, Kp196 and KPCEU1 genomes were compared, together with other publicly available *K. pneumoniae* ST11 genomes from Brazil (Fig. 1), in order to assess their genetic relationship. Moreover, the main differences between Kp196 and KPCEU1 resistomes were revealed (Fig. 2), contributing to insights into *K. pneumoniae* high-risk clones in the eastern Amazon Region. The genetic reconstruction revealed that Kp196 and KPCEU1, despite belonging to ST11, were not clonal (Fig. 1). In fact, several lineages from ST11 had already been revealed in Brazil.^6^ Moreover, concerning the acquired resistome, KPCEU1 genome presented an expressive arsenal of resistance genes, despite its environmental nature. It shares several genes with Kp196 (*oqxAB*, *bla*_CTX-M-15_, *bla*_OXA-1_, *bla*_SHV-11_, *sul*, *aph(3’)-I* and *fosA*), but also presented a particular set of genes involved with resistance to several antibiotic classes such as quinolones (*qnrB19, aac(6’)-Ib-cr5*), chloramphenicol (*catB3*), rifampin (*arr-3*), quaternary ammonium compounds (*qacEΔ1*), macrolides (*mphA*, *mrxA*), chromate (*chrA*), and β-lactams including carbapenems (*bla*_KPC-2_) (Fig. 2). Interestingly, the KP196 and KPCEU1 carried distinct carbapenemase-coding genes. While KPCEU1 harboured the *bla*_KPC-2_, highly disseminated and prevalent in Brazil and in the world, KP196 carried the *bla*_NDM-1_, which is not so frequent. Several of these genes were found in the context of a class 1 integron embedded in an IS*6100* backbone (*intI1*-*aac(6’)-Ib-cr5*-*bla*_OXA-1_-*catB3*-*arr-3*-*qacEΔ1/sul1*-*chrA*-*padR*-IS*6100 tnpA*-*mphR*-*mrxA*-*mphA*).

Regarding the intrinsic mechanisms in Kp196, mutations were observed in genes involved with resistance to fluoroquinolones (*gyrA*, *parC*), colistin (*mgrB*, *arnT, phoQ*), tigecycline (*ramR*), and multiple drugs including carbapenems and cephalosporins (*acrB*, *ompK35*, *ompK36*, and *ompK37*). Ciprofloxacin is effective and widely used for treating ESBL-producing *K. pneumoniae* infections. The Kp196 presented substitutions in the quinolone resistance-determining region (QRDR) of GyrA (S83I) and ParC (S80I), which are involved with ciprofloxacin resistance emergence in *K. pneumoniae*.

Colistin resistance in *K. pneumoniae* is mainly associated with modifications in *pmrAB*, *phoPQ*, *mgrB,* and *arnT* genes.^21^ Among these genes, substitutions were found in the deduced protein of ArnT (M114L, V117I, and R372K), and in PhoQ (D150G). Besides, the *mgrB* was disrupted by IS*Kpn25* at the nucleotide position 133, leading to the production of a truncated and inactive MgrB protein, probably contributing to Kp196 colistin resistance due to *phoPQ* derepression.^21^ Interestingly, this same alteration was previously found in colistin-resistant ST258 *K. pneumoniae* from Greece and Brazil,^22^ indicating that this region might be a hotspot for IS*Kpn25* insertion. This IS additionally carried *bla*_TEM-1_, *aac(3)-IId*, and a complete restriction modification system (RMS), also contributing to β-lactams and aminoglycosides resistance, and to host protection from foreign DNA infection. Therefore, the Kp196 colistin resistance could be associated with the accumulation of multiple alterations in chromosomal genes (*mgrB*, *arnT,* and *phoQ*). In this case, even upon restoration of the canonical function by reversal mutations in one of these genes, Kp196 would retain the colistin resistance (Table II).

**TABLE II:** Acquired and intrinsic resistance mechanisms involved with the Kp196 PDR phenotype

Horizontally acquired resistance genes	Antibiotic classes	Resistance alterations in chromosomal genes
*aacA4* *aac(3)-IId* *aph(3’)-VI* *aadA1* *strAB*	*Aminoglycosides*	X
*tet(D)*	*Tetracyclines*	AcrB _(S966A)_ *ramR*_(V19A, T119H; gene disruption by 14-bp deletion at nt 330-373)_ *ompK35*_(frameshit by a 1-bp deletion C338Δ)_ *ompK36*_(gene disruption by a 523-bp deletion at nt164 - 687)_ *ompK37*_(gene inactivation by several missenses mutations and insertions)_
*catB3*	*Phenicol*	
*bla*_NDM-1 (except for monobactam)_ *bla*_CTX-M-15 (except for carbapenems and penicillins + β-lactam inhibitors)_ *bla*_SHV-11,_ *bla*_OXA-1,_ *bla*_OXA-9,_ *bla*_TEM-1 (only to penicillins and some narrow spectrum β-lactams)_	*Cephalosporins*	
	*Carbapenems*	
	*Penicillins and Penicillins* + *β-lactam inhibitors*	
	*Monobactam (Aztreonam)*	
*qnrS1* *qnrB1* *oqxAB*	*Fluoroquinolones*	GyrA _(S83I)_ ParC _(S80I)_ OqxR _(V130A)_ RarA _(Q172R, V191I)_
*dfrA14* *sul2*	*Folate pathway inhibitor*	X
*fosA5*	*Phosphonic acid*	X
X	*Glycylcyclines (tigecycline)*	AcrB _(S966A)_ *ramR*_(V19A, T119H; gene disruption by 14-bp deletion at nt 330-373)_
X	*Polymyxins*	*mgrB*_(gene disruption by ISKpn25 at nt 133)_ PhoQ _(D150G)_ ArnT _(M114L, V117I, R372K)_

In spite of several tigecycline resistance mechanisms already described, *K. pneumoniae* tigecycline-resistant strains remain rare.^22^ Since Kp196 was resistant to tigecycline and lacks the *tetX* gene, a plasmid-borne gene involved with resistance to tetracyclines, we searched for alternative non-enzymatic mechanisms. Among these mechanisms, efflux pump overexpression (*acrAB* and *oqxAB*) due to alterations in their regulatory genes (*ramR*, *ramA*, *soxR*, *soxS*, *marA*, *marR*, *acrR*, *oqxR*, *rarA*) is the most common.^23^ From all the aforementioned regulatory genes, only *ramR* (*ramA* repressor), *oqxR* and *rarA* (*oqxAB* repressor and activator, respectively) were altered in Kp196. The RamR presented two amino acid modifications (V19A and T119H) and a 14 bp-deletion downstream the nt 330 was present in this gene, leading to a frameshift. This *in-block* deletion probably generated an inactivated RamR, resulting in *ramA* derepression and, consequently, to *acrAB* overexpression. The substitutions found in RarA (Q172R and V191I) have not been described yet, while the OqxR presented the V130A alteration that had already been found in tigecycline-susceptible strains.^23^ Therefore, the *ramR* alterations were probably the main tigecycline and multidrug resistance determinant in Kp196 (Table II).

Kp196 harboured the S966A AcrB variant, which is involved with the increment of drug transport efficiency, conferring an increased ability to persist/resist its substrate antibiotics when overexpressed.^24^ Since *acrAB* is also involved with resistance to other tetracyclines, fluoroquinolones, erythromycin, β-lactams, chloramphenicol, and also carbapenems,^25^ ^,^ ^26^ ^,^ ^27^ the *acrAB* overexpression with an enhanced-function AcrB variant may also contribute with the remarkable Kp196 multidrug resistance phenotype.

In *K. pneumoniae*, loss of the two major outer membrane porins OmpK35 and OmpK36 enhances the multidrug resistance in ESBL-producing strains, increasing resistance to carbapenems, broad-spectrum cephalosporins, fluoroquinolones, tetracycline, and chloramphenicol.^28^ In Kp196, the *ompK35* suffered a deletion at nucleotide 338 resulting in a frameshift, while an *in-block* deletion from nucleotide 164 to 687 disrupted *ompK36*. The *ompK37* is normally expressed only in *ompK35-36*-deficient strains, slightly influencing carbapenem resistance.^28^ However, in addition to *ompK35*/*36*, the *ompK37* of Kp196 was also altered, presenting a set of SNPs and insertions along the gene that could lead to a defective porin. Therefore, all three *K. pneumoniae* major porins were probably inactivated in Kp196, which could significantly contribute to multidrug resistance in this strain. Finally, considering the clinical relevance of carbapenem resistance, this study stressed the multifactorial and overrepresented mechanisms in Kp196, which comprised the presence of *bla*_NDM-1_ and alterations of several intrinsic genes, such as *acrAB*, *ompK35-36-37*. Interestingly, the unique genomic studies on CC258 *K. pneumoniae* PDR strains in Brazil demonstrated a different resistome composition compared to Kp196, considering both the intrinsic and acquired resistance determinants involved with PDR manifestation.^4^ ^,^ ^5^ Besides, in both studies, the PDR phenotype was mainly due to the presence of acquired resistance genes.

The genome of the environmental ST11 KPCEU1 strain was screened for all the aforementioned genes involved with intrinsic resistance in Kp196. Interestingly, despite their non-clonal nature (Fig. 1) and carrying a different acquired resistome as demonstrated above, KPCEU1 harboured the same polymorphisms in the housekeeping genes (*anrT*, *phoQ*, *rarA*, *ompK37*, *gyrA*, *parC*, *marA*, *marR*) as found in Kp196. The exception was the presence of canonical versions of *ramR*, *mgrB*, and *ompK35*, and the loss of *ompK36* in KPCEU1.

Previous studies had already demonstrated that *K. pneumoniae* strains from the same lineage, mainly those from pandemic lineages such as ST11, can circulate among natural and clinical environments under distinct adaptation pressures that differentially shape the bacterial genomic features.^29^ It has been assumed that the natural environment could promote genetic diversification concerning efflux and other mechanisms involved with cell metabolism and physiology, while the clinical context would drive the acquisition of antibiotic resistance genes and virulence traits.^29^ However, our results showed a different scenario, since the KPCEU1 environmental strain presented a remarkable resistome concerning acquired resistance genes. Moreover, the acquired resistome of the clinical and environmental ST11 strains was quite different (Fig. 2), while the resistome related to intrinsic genes was almost identical between Kp196 and KPCEU1. The *in silico* analysis revealed a different set of plasmid replicons between Kp196 (IncFIB and IncR) and KPCEU1 (IncN), which could explain the unique acquired resistome observed in each genome.

*In conclusion* - Here, the genome of a clinical *K. pneumoniae* strain belonging to the pandemic ST11 lineage and presenting a PDR phenotype was revealed in the eastern Amazon Region. In parallel, we demonstrated that the unique other *K. pneumoniae* available genome from Maranhão (also belonging to ST11), carried an expressive resistome despite its environmental origin. In this way, this study contributed to genomic and epidemiological information concerning a *K. pneumoniae* high-risk lineage in an underrepresented Brazilian region. Interestingly, both the clinical (Kp196) and environmental (KCEU1) genomes presented a more stable resistome, since multiple mutations in chromosomal genes, which are not easily lost as the acquired resistance determinants, were verified and could importantly contribute to the observed Kp196 PDR phenotype.
